# Supplementary material for: Evaluating the Necessity and Impact of Cardiac Imaging on Breast Cancer Care in Northwestern Ontario
Source: Cancers (Basel). 2025 Jun 8;17(12):1909. doi: 10.3390/cancers17121909 (PMC12191271; doi:10.3390/cancers17121909)
Supplement: Supplementary file 1 [file cancers-17-01909-s001.zip › CO - Supplementary Tables.pdf]

Supplementary Table S1: Medical History Before Starting Treatment

Comorbidity	Total (n = 93)	Cohort A (n = 3)	Cohort B (n = 60)	Cohort C (n = 30)
**Pre-treatment/earliest ECOG score**				
0, n (%)	53 (56.99)	2 (66.67)	34 (56.67)	17 (56.67)
1, n (%)	32 (34.41)	1 (33.33)	19 (31.67)	12 (40.00)
2, n (%)	6 (6.45)	0 (0.00)	6 (10.00)	0 (0.00)
Unknown, n (%)	2 (2.15)	0 (0.00)	1 (1.67)	1 (3.33)
**Pre-treatment/earliest Karnofsky score**				
Known, n (%)	0 (0.00)	0 (0.00)	0 (0.00)	0 (0.00)
Unknown, n (%)	93 (100.00)	3 (100.00)	60 (100.00)	30 (100.00)
**Pregnancy**				
Yes, n (%)	0 (0.00)	0 (0.00)	0 (0)	0 (0.00)
No, n (%)	93 (100.00)	3 (100.00)	60 (100)	30 (100.00)
**Diabetes**				
Yes, n (%)	9 (9.68)	0 (0.00)	7 (11.67)	2 (6.67)
No, n (%)	83 (89.25)	3 (100.00)	53 (88.33)	27 (90.00)
Borderline, n (%)	1 (1.08)	0 (0.00)	0 (0.00)	1 (3.33)
**Hypertension**				
Yes, n (%)	44 (47.31)	2 (66.67)	26 (43.33)	16 (53.33)
No, n (%)	49 (52.69)	1 (33.33)	34 (56.67)	14 (46.67)
**High cholesterol**				
Yes, n (%)	18 (19.35)	1 (33.33)	13 (21.67)	4 (13.33)
No, n (%)	75 (80.65)	2 (66.67)	47 (78.33)	26 (86.67)
**Smoking status**				
Current, n (%)	13 (13.98)	0 (0.00)	8 (13.33)	5 (16.67)
Former, n (%)	31 (33.33)	0 (0.00)	20 (33.33)	11 (36.67)
No, n (%)	49 (52.69)	3 (100.00)	32 (53.33)	14 (46.67)
**Coronary artery disease**				
Yes, n (%)	8 (8.60)	0 (0.00)	6 (10.00)	2 (6.67)
No, n (%)	85 (91.40)	3 (100.00)	54 (90.00)	28 (93.33)
**Heart failure**				
Yes, n (%)	0 (0.00)	0 (0.00)	0 (0.00)	0 (0.00)
No, n (%)	93 (100.00)	3 (100.00)	60 (100.00)	30 (100.00)
**Valvular disease**				
Yes, n (%)	2 (2.15)	0 (0.00)	1 (1.67)	1 (3.33)
No, n (%)	91 (97.85)	3 (100.00)	59 (98.33)	29 (96.67)
**Atrial fibrillation**				
Yes, n (%)	3 (3.23)	0 (0.00)	2 (3.33)	1 (3.33)
No, n (%)	90 (96.77)	3 (100.00)	58 (96.67)	29 (96.67)
**COPD**				
Yes, n (%)	9 (9.68)	0 (0.00)	7 (11.67)	2 (6.67)

No, n (%)	84 (90.32)	3 (100.00)	53 (88.33)	28 (93.33)
**Peripheral vascular disease**				
Yes, n (%)	1 (1.08)	0 (0.00)	1 (1.67)	0 (0.00)
No, n (%)	92 (98.92)	3 (100.00)	59 (98.33)	30 (100.00)
**Stroke/TIA**				
Yes, n (%)	1 (1.08)	0 (0.00)	1 (1.67)	0 (0.00)
No, n (%)	92 (98.92)	3 (100.00)	59 (98.33)	30 (100.00)
**Chronic kidney disease**				
Yes, n (%)	0 (0.00)	0 (0.00)	0 (0.00)	0 (0.00)
No, n (%)	93 (100.00)	3 (100.00)	60 (100.00)	30 (100.00)
**Previous breast cancer**				
Yes, n (%)	4 (4.30)	0 (0.00)	2 (3.33)	2 (6.67)
No, n (%)	89 (95.70)	3 (100.00)	58 (96.67)	28 (93.33)
**Previous other cancer**				
Yes, n (%)	11 (11.83)	0 (0.00)	8 (13.33)	3 (10.00)
No, n (%)	82 (88.17)	3 (100.00)	52 (86.67)	27 (90.00)
**Previous anthracycline**				
Yes, n (%)	2 (2.15)	0 (0.00)	2 (3.33)	0 (0.00)
No, n (%)	91 (97.85)	3 (100.00)	58 (96.67)	30 (100.00)
**Previous radiation**				
Yes, n (%)	4 (4.30)	0 (0.00)	1 (1.67)	3 (10.00)
No, n (%)	89 (95.70)	3 (100.00)	59 (98.33)	27 (90.00)

Supplementary Table S2: Characteristics of cardiac imaging studies

Characteristic	Overall (n = 93)	Cohort A (n = 3)	Cohort B (n = 60)	Cohort C (n = 30)
**Method**				
Echocardiogram, n (%)	16 (17.20)	1 (33.33)	11 (18.33)	4 (13.33)
MUGA, n (%)	77 (82.80)	2 (66.66)	49 (81.67)	26 (86.67)
CMR, n (%)	0 (0.00)	0 (0.00)	0 (0.00)	0 (0.00)
**Study time-point**				
Baseline, n (%)	59 (63.44)	0 (0.00)	51 (85.00)	8 (26.67)
During chemotherapy, n (%)	10 (10.75)	0 (0.00)	6 (10.00)	4 (13.33)
Between chemotherapy and trastuzumab start, n (%)	5 (5.38)	0 (0.00)	3 (5.00)	2 (6.67)
After chemotherapy	19 (20.43)	3 (100.00)	N/A	16 (53.33)

completion and trastuzumab start, n (%)				
**Referring physician**				
Oncologist, n (%)	87 (93.55)	3 (100.00)	55 (91.67)	29 (96.67)
Family physician, n (%)	3 (3.26)	0 (0.00)	3 (5.00)	0 (0.00)
Cardiologist, n (%)	1 (1.08)	0 (0.00)	1 (1.67)	0 (0.00)
Breast surgeon, n (%)	1 (1.08)	0 (0.00)	0 (0.00)	1 (3.33)
Other, n (%)	1 (1.08) [a]	0 (0.00)	1 (1.67) [a]	0 (0.00)
**Ordering mechanism**				
Routine, n (%)	84 (90.32)	3 (100.00)	52 (86.67)	29 (96.67)
Prompted by clinical factors, n (%)	9 (9.68)	0 (0.00)	8 (13.33)	1 (3.33)
**Average LVEF**				
Mean (SD)	62.32 (9.32)	58 (9.90)	63.65 (8.91)	60.15 (9.88)
Range	29-80	51-65	29-80	38-75

[a] critical care physician
